# Biorefineries: Achievements and challenges for a bio-based economy

**DOI:** 10.3389/fchem.2022.973417

**Published:** 2022-11-10

**Authors:** Francisco G. Calvo-Flores, Francisco J. Martin-Martinez

**Affiliations:** ^1^ Grupo de Modelizacion y Diseño Molecular, Departamento de Quimica Organica, Universidad de Granada, Granada, Spain; ^2^ Department of Chemistry, Swansea University, Swansea, United Kingdom; ^3^ Department of Materials Science and Engineering, Massachusetts Institute of Technology, Cambridge, MA, United States

**Keywords:** biomass valorisation, biorefineries, biotechnological platform, thermochemical platform, circular economy, lignin, biomass

## Abstract

Climate change, socioeconomical pressures, and new policy and legislation are driving a decarbonization process across industries, with a critical shift from a fossil-based economy toward a biomass-based one. This new paradigm implies not only a gradual phasing out of fossil fuels as a source of energy but also a move away from crude oil as a source of platform chemicals, polymers, drugs, solvents and many other critical materials, and consumer goods that are ubiquitous in our everyday life. If we are to achieve the United Nations’ Sustainable Development Goals, crude oil must be substituted by renewable sources, and in this evolution, biorefineries arise as the critical alternative to traditional refineries for producing fuels, chemical building blocks, and materials out of non-edible biomass and biomass waste. State-of-the-art biorefineries already produce cost-competitive chemicals and materials, but other products remain challenging from the economic point of view, or their scaled-up production processes are still not sufficiently developed. In particular, lignin’s depolymerization is a required milestone for the success of integrated biorefineries, and better catalysts and processes must be improved to prepare bio-based aromatic simple molecules. This review summarizes current challenges in biorefinery systems, while it suggests possible directions and goals for sustainable development in the years to come.

## 1 Introduction

Complying with net-zero targets, reducing greenhouse gas emissions, and transitioning to an environmentally, economically, and socially sustainable development are complex and colossal challenges that require a highly efficient and cost-effective biorefinery system, among other interrelated factors. Biorefineries must integrate with current infrastructure, while enabling the transformation of a wide range of biological feedstocks and waste into a portfolio of bio-based materials and specialty chemicals, whose cost do not exceed the cost of their petrochemical counterparts and whose performance is proven to be at least as good as that of the petrochemical equivalents. For centuries, the traditional uses of biomass have been limited to animal feeding, energy generation, i.e., heating or cooking, and some engineering and commodity applications, including wood for construction and furniture and natural fibers for textiles. Traditional processing techniques also enabled the obtention of biomass-derived products such as vegetable oils or flours using milling, essences, and fragrances *via* steam distillation, valuable platform chemicals through liquid–liquid extraction, and yogurt, kefir, wine, or beer, from fermentation processes. Despite its abundance, applicability, and use, the list of substances, materials, and key building blocks available from biomass to produce fine and specialty chemicals is still quite limited compared to those provided by the petrochemical industry. Furthermore, the lack of cost-efficient scaled-up processes and the lower performance of many biorefinery products *versus* petrochemical alternatives have kept our economy still largely based on fossil sources.

Following the United Nations (UN) definitions of sustainability, it is our responsibility to meet the needs of the present without compromising the ability of future generations to meet their own needs. Nowadays, among the 17 sustainable development goals (SDGs) highlighted by the UN, there are at least six goals, i.e., clean water and sanitation, affordable and clean energy, industry innovation and infrastructure, sustainable cities and communities, responsible consumption and production, and climate action, that are heavily dependent on two enormous challenges:

1) Drastically and rapidly reducing our atmospheric emissions of greenhouse gasses
2) Replacing crude oil as a non-renewable source, while maintaining a sustainable development in the longer term

Addressing the first challenge requires, among other actions, the development of more efficient renewable energy platforms, as well as the electrification of the transportation sector, powered by an energy grid with zero CO_2_ emissions. The second challenge calls for the development of technologies that shift our current material sources to more renewable raw materials, ideally from waste resources, while reducing many countries’ overdependency on fossil fuel imports or extraction. To this end, biorefineries emerge as a critical solution to co-produce materials, sustainable fuels, and platform chemicals from a great diversity of non-eatable biomass feedstocks.

There are four main features that are commonly used as criteria for the classification of biorefinery systems: feedstock, processes, platforms, and products. In the following, we will briefly discuss the feedstock and process and further expand the discussion on biorefinery platforms and products through the subsequent sections.

Concerning the feedstock, the definition of biomass depends on the source, and it is open to discussion about what to include or not to include under the consideration of biomass. In a very general way, it is any organic matter, derived from living, or recently living animals or plants such as crops, as well as the waste derived from them, or other residues from municipal wastes, wastewater treatment, and feedstock. Common feedstocks include grasses, starch crops from wheat and maize, sugar crops from beet and cane, lignocellulosic materials from wood, crops or residues, oil crops, algae and seaweeds, and many other organic wastes from industrial, agriculture, and livestock activities, as well as commercial and post-consumer waste (Jong et al., 2012). In biorefineries, this feedstock can be obtained from

1) A dedicated production to obtain sugar and starch, lignocellulosic materials, oils, and marine biomass
2) Residual biomass formed by organic residues from urban waste, sludges, manure, industrial lignin residues, and oil-based residues

The main pre-processing and processing technologies used in biorefinery are as follows:

1) Mechanical processes, e.g., pressing, fractionation, and size reduction
2) Chemical processes, e.g., acid hydrolysis, oxidations, and esterification
3) Thermochemical processes, e.g., hydrothermal processing, pyrolysis, and gasification
4) Biochemical processes, e.g., fermentation

The mechanical and chemical processes are usually used as pre-processing techniques to breakdown biomass before getting into a thermochemical or biochemical process. These methods can be combined in different ways depending on the feedstock, the platform, and the intended application, for example, biofuels, chemicals, or a combination of both (Cherubini et al., 2009).

In the following sections, this review summarizes the origins of the biorefinery concept, the biorefinery platforms and technologies, some biorefinery products, and some of the current challenges and future directions in biorefinery development.

## 2 The biorefinery concept

It is unquestionable that the industrial revolution shaped the foundations of our current society for the good and bad. At first, this revolution was powered by coal as the primary energy source and it evolved into a crude oil-based economy during the following decades. We are now in a new crucial period, in which our society is involved in a mandatory but challenging transition to a bioeconomy based on renewable sources of energy and materials. In this new paradigm, which is driven by environmental, socioeconomical, and legislative reasons, the concept of biorefinery has emerged as one of the strongest enablers to a new economy that shies away from fossil fuels.

The biorefinery concept arose during the late 1990s, in parallel with the birth of green chemistry (Anastas and Warner, 2000). The United States Department of Energy defined biorefineries as processing plants where biomass feedstock is converted and/or extracted into a spectrum of valuable products to produce fuels and high-value chemicals (Kamm and Kamm, 2004). In fact, the seventh principle of green chemistry encourages the use of renewable feedstocks rather than those from non-renewable sources, such as coal or crude oil. Thus, biorefineries are the sustainable alternative to the traditional refineries of the petrochemical industries (Wagemann and Tippkötter, 2019).

### 2.1 Biorefinery technological platforms

In the petrochemical industry, the concept of the platform refers to the crude oil fractionation into many key intermediates that are further processed to obtain energy and/or chemicals. Similarly, in a biorefinery platform, biomass is fractionated into a set of key intermediate chemicals of varying purity that are further processed. The platforms represent the link between the raw materials and the final products, and they are the most important feature in biorefineries. According to the technology used in the biomass conversion, biorefineries implement two main types of platforms, which can be used in a single way or combined: thermochemical platforms and biotechnological platforms. Taking into count the diversity of biomass and the availability of feedstocks, it is possible that different types of biomasses are processed through the same platform, or that a biorefinery implements different platforms at the same time.

Moreover, according to the number of feedstocks processed and the number of products produced, biorefineries are classified into *phase I*, *phase II*, and *phase III* (Wagemann and Tippkötter, 2019), (Oecd and OECD, 2018), (Cherubini et al., 2009). Thus, *phase I* biorefineries use a single feedstock material to prepare a primary product, such as biodiesel biorefineries that produce biofuel from vegetable oils (Inamuddin et al., 2021), (Caullet and Le Nôtre, 2015) or from algae (Inamuddin et al., 2021; Konur, 2021). *Phase II* biorefineries carry out a set of processes to produce different products from a single feedstock material, for example, processing grain to produce ethanol and carbon dioxide (Kamm and Kamm, 2004). *Phase III* biorefineries produce multiple types of products from multiple feedstock materials through a diverse processing technology (Fernando et al., 2006). In the case of *phase III* biorefineries, there are four additional subgroups that are commonly mentioned:

1) Whole-crop biorefinery (Koutinas et al., 2022), (Thongchul et al., 2022): Raw crops such as wheat or corn are used as a unique feedstock material to produce value-added products such as chemical building blocks, pharmaceutical products, textiles, plastics, lubricants, and biofuels
2) Green biorefinery (McEniy and O’ Kiely, 2014): Natural-wet biomass such as cereals or grass are processed and transformed into marketable chemicals and fuels.
3) Lignocellulosic biorefinery (De Bhowmick et al., 2018): Lignocellulosic biomass is converted into value-added products such as bio-oil, biochar, or other bio-based chemicals.
4) Two-platform concept biorefinery (De Bhowmick et al., 2018; Buck et al., 2020): Both thermochemical and biochemical conversions take place in an integrated design to produce valuable products and fuels.

The obtention of valuable chemicals, fuels, or any other product often requires a set of physical or chemical pre-treatments depending on the biomass type to obtain optimal results (Cantero et al., 2019). Common physical pretreatments consist of mechanical separations, milling, liquid–liquid extractions, or distillations which basically do not change the chemical composition and structure of the feedstock components, but help to transform the biomass in the subsequent processing steps. Chemical pretreatments consist of a set of single or combined reactions such as hydrolysis, hydrogenation, oxidation, or pulping that slightly modify biomass to provide a better transformation afterward. Table 1 summarizes the classification of biorefinery evolution based on feedstock and final products.

**TABLE 1:** Types of biorefineries according to number of feedstocks processed and number of products produced.

Type of biorefinery	Type of feedstock	Final product
Phase I	Single feedstock	Primary products
Phase II	Single feedstock	Several products
Phase III	Multiple feedstocks	Multiple products

### 2.2 Thermochemical platform

**FIGURE 1:**
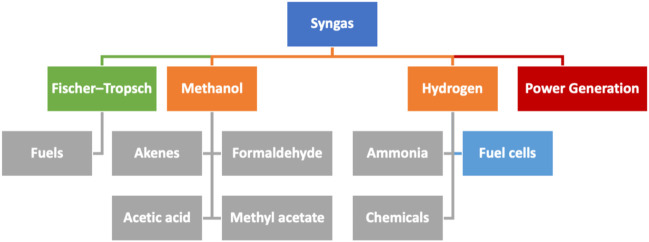
Schematic representation of the possible routes for valorizing syngas (in blue). It includes the Fischer–Tropsch process (in green) to produce fuels (in gray), power generation (in red), and the main intermediates (in orange) to produce chemicals (in gray) or being applied in fuel cells (in light blue).

In thermochemical platforms, biomass undergoes high-temperature processing, many times combined with high pressures, and with or without the presence of solvents and catalysts (Salan, 2017). These platforms are mostly focused on the production of biofuels, although there are some cases in which chemicals are also produced at high temperature in an oxygenic or anoxygenic environment (International Energy Agency, 2016). The main technologies applied in thermochemical platforms are as follows:

1) Combustion (Mandø, 2013): It is the simplest form of thermochemical conversion of biomass. It involves the oxidation of biomass in an oxygen-rich ambient to produce energy. It is extensively applied given its low cost, high reliability, and its easy integration with the pre-existing infrastructures on both large- and small-scale levels (Luo and Zhou, 2012). For example, the pulp and paper industry has traditionally used the residual lignin byproduct from the pulping process as a waste to produce energy by combustion (Bajwa et al., 2019).
2) Carbonization (Amer and Elwardany, 2020), (Ronsse et al., 2015): It is one of the oldest biomass conversion processes known. It is a thermochemical process to increase the carbon content by the volume of biomass through thermochemical decomposition. It is commonly applied to the production of charcoal-like bio-based materials at temperatures in the range of 500°C–900°C, and in a poor oxygen atmosphere, to avoid combustion.
3) Gasification (Heidenreich et al., 2016): It is a thermochemical process that applies higher temperatures than carbonization (from 800 to 1,200°C) in the presence of pure oxygen or air under stoichiometric amounts to avoid total oxidation and combustion reactions in order to produce a mix of light gasses, mainly hydrogen, carbon monoxide, carbon dioxide, and methane. The gases produced can be used directly as fuel or they can be separated and transformed into other products through chemical or enzymatic transformations. The mixture of carbon monoxide and hydrogen is known as synthesis gas, that is, syngas. Although the preparation of syngas has been carried out from coal for more than 100 years (Reyes et al., 2003), it is currently produced from natural gas or biomass. Syngas is converted into liquid hydrocarbons and oxygenated compounds such as alcohols, aldehydes, and others, for example, by the well-known Fischer–Tropsch process (Fischer-Tropsch Synthesis, 2010) (Figure 1).
4) Pyrolysis (Madár et al., 2018): It is a thermochemical process to convert biomass by external heating under an inert atmosphere. The processing conditions drive the decomposition of biomass polymer chains to produce bio-oils, gasses, and biochar, that is, a solid carbon-based material with a highly porous structure (Benedetti et al., 2018). Pyrolysis is a well-established method to produce high-value products from biomass (Wang et al., 2020).
5) Hydrothermal processing (Grande et al., 2021): This is a thermochemical process for the conversion of several feedstocks (Elliott et al., 2015) into biocrude oils (Grande et al., 2021) (hydrothermal liquefaction) or hydrochar (hydrothermal carbonization). It heats wet biomass under subcritical water conditions (Xue et al., 2016). Typical conditions ranges from 200 to 400°C and pressures from 20 to 200 bars, having subcritical water to act as both solvent and catalyst for the decomposition of biomass.

### 2.3 Biotechnological platforms

Biotechnological platforms are based on the enzymatic conversion of biomass, that is, fermentations (B et al., 2016). The conversion process occurs under milder conditions than in the thermochemical platforms and they can be performed under aerobic or anaerobic conditions, or under combinations of them. These platforms are mostly focused on the preparation of high-value chemicals, rather than biofuels.

**FIGURE 2:**
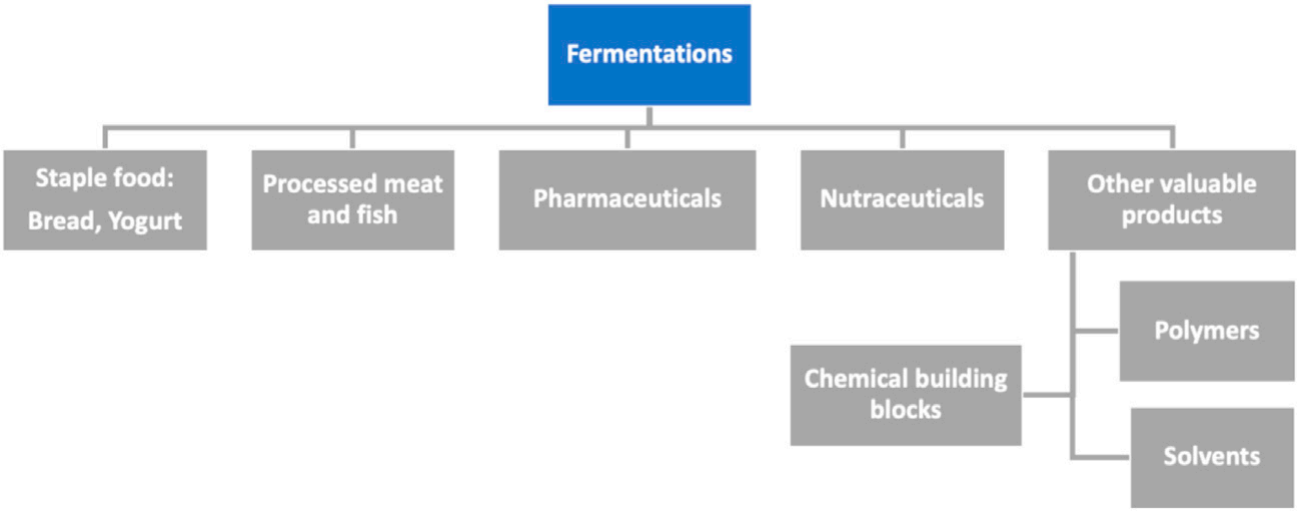
Schematic representation of the main areas (in gray) in which fermentation processes (in blue) are traditionally applied.

The term “ferment” comes from the Latin word *fervere*, which means to leaven. Natural fermentation is a process that has been used for centuries to produce beer, wine and other beverages, yogurt, kombucha, cheese, bread, or cured salami, to mention a few. Currently, the term is extended to any chemical transformation provided by microorganisms or pure enzymes (Figure 2).

Food and pharmaceutical industries have used enzymatic transformations for decades. In the food industry, microorganisms and enzymes are used for the preparation, not only of traditional products described before, but also other food-grade bio-preservatives, i.e., nutraceuticals substances (Chisti, 2020), which has a physiological benefit or provide protection against diseases (Nasri et al., 2014). The pharmaceutical industry uses fermentations to produce antibiotics, insulin, hormones, and vaccines (Walsh, 2013), in combinations with other strategies to improve the efficiency, the selectivity, and the versatility in enzymatic transformations (Baweja et al., 2016).

**FIGURE 3:**
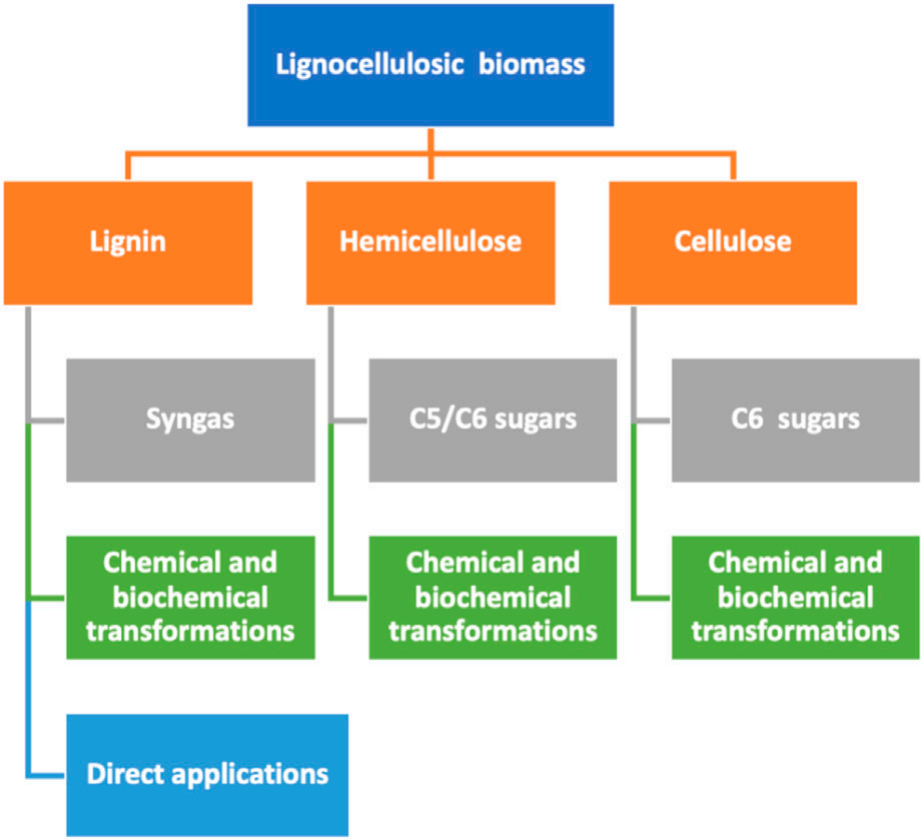
Schematic representation of the possible routes for valorizing lignocellulosic biomass (in blue). It includes the main intermediates (in orange) to produce valuable products (in gray) or being directly applied (in light blue).

Although the differences between different groups of biotechnological platforms in biorefineries are sometimes subtle, they can be generally grouped as described in the following:

a) Syngas platform: As previously discussed, the main constituents of syngas are carbon monoxide and hydrogen, commonly produced through gasification. These intermediates can be subjected to a broad range of chemical and microbial transformations, to give gaseous and liquid fuels and many other fine chemicals (Dahmen et al., 2017). Despite being known for many years, syngas production from biomass is receiving increasing attention. It is a quick flexible process regarding both the biomass feedstock utilized and the possible final products that can be produced through it. The rather low carbon content of biomass (15–50%), compared to coal (60–85%), and a high moisture content in waste biomasses are fundamental challenges for the scale-up of this technology. In addition, the great heterogeneity of biomass considerably increases transportation and production costs (Frazão and Walther, 2020). Nevertheless, it is still a very promising alternative to produce hydrogen from renewable sources. The reported estimations about the yields and costs of hydrogen production from renewable feedstock point out biomass gasification as the best avenue for technology development (Lepage et al., 2021).
b) Biogas platform: The term biogas describes the mixture of gasses obtained from the anaerobic digestion of biomass, that is, mostly methane and carbon dioxide (Bio-Based Chemicals: A 2020 Update, 2020). Methane can be used for heating or power generation within the biorefinery, but it can be also incorporated into the conventional natural gas distribution system, compressed for its use as fuel transportation in light and heavy-duty vehicles, or as a precursor of bio-based chemicals such as ammonia, methanol, oxo chemicals, acetylene, and hydrogen cyanide. (Speight, 2022).
c) C5/C6 carbohydrates platform: Valorization of five-carbon (C5) and six-carbon (C6) sugars from several sources is probably the best-developed branch in current biorefineries. These C5 and C6 carbohydrates can be accessed from the depolymerization of starch or hemicellulose and cellulose. To increase the yield and speed up biomass decomposition, the feedstock is typically subjected to a first pre-treatment process, which is then followed by an either chemical or enzymatic treatment that finally liberates simple sugars to be transformed into fuels and chemicals. In this platform, the two feedstocks mostly utilized are:
  1) Carbohydrates from grain and related crops: Such feedstocks are mainly composed of C6 carbohydrate polymers such as cellulose or starch, disaccharides such as sucrose, and other simpler C6 carbohydrates. In case of C6-based polymers, either chemical or enzymatic hydrolysis is performed to obtain the monosaccharide C6 units that are then usually transformed into ethanol or other valuable molecules through fermentation.
  2) Carbohydrates from lignocellulosic biomass: Lignocellulosic biomass refers to non-edible biomasses that are obtained from sources such as agricultural and forestry wastes, energy crops (switchgrass), or woody residue (poplar) (Merklein et al., 2016). This is the most cost-efficient and highly renewable natural resource worldwide (Qian, 2014), and it constitutes a ubiquitous source of carbohydrates that can be isolated and transformed into valuable chemicals within biorefineries. The three major components of lignocellulosic biomass are lignin, cellulose, and hemicellulose (Figure 3). The amount of each component within the lignocellulosic structure is very diverse. Depending on the biomass source, the composition varies, with a variable proportion of carbohydrates between 60% and 70% (Chen, 2014). Cellulose is the major component, and hemicellulose is about a third of the total biomass dry weight (Frassoldati and Ranzi, 2019). As it is well known, cellulose is the most abundant structural polysaccharide in plants. It is formed by glucose units (C6), and it can be subjected to both the chemical and the enzymatic processes to produce glucose molecules. Hemicellulose is a more complex copolymer formed by linear or branched chains of C6 carbohydrates, such as galactose, mannose, glucose, 4-O-methyl glucuronic acid, and galacturonic acid residues, in combination with C5 units, mainly xylose and arabinose. Depolymerization of hemicellulose occurs more quickly than that for cellulose, and the C5 sugars derived from the process are generally more reactive than the glucose obtained from cellulose (Kobayashi and Fukuoka, 2013). Depending on the isolation method for separating hemicellulose from biomass, different types of products and materials are obtained. In fact, hemicellulose is a starting material for the preparation of biofuels (Varejão and Varejão, 2022), valuable molecules (Sun et al., 2021) of technical bioproducts (Huang et al., 2021).

d) Lignin platform: Lignin is the second most abundant biopolymer in nature after cellulose, and the first source of aromatic chemical moieties, excluding crude oil. Hence, lignin’s relevance as a renewable raw material is unquestionable, and in fact crucial for the success of integrated biorefineries in the context of a bio-based economy. However, after countless efforts and research devoted to efficient and scaled-up lignin depolymerization processes for years, many challenges still stand ahead. Lignin is a randomly cross-linked, aquiral polymer, formed mainly by three units with a phenolic core, i.e., p-coumaryl alcohol, coniferyl alcohol, and sinapyl alcohol, also known as monolignols (Calvo-Flores and Dobado, 2010). These monolignols are interconnected and additionally linked to some carbohydrate chemical moieties to form an amorphous structure that it is challenging to characterize, isolate, and depolymerize. Furthermore, there is no unique structure for lignin, but it differs in its origin, e.g., hardwood, softwood, or different herbaceous plants. On the other hand, under the umbrella of industrial or commercial lignins, there are also some other lignin types that are isolated from industrial processes, mostly from the pulp and paper industry (Calvo-Flores et al., 2015). In fact, approximately 50 million tons of lignin are currently produced annually as a byproduct of the pulp and paper industry (Bajpai, 2016). Attending to the isolation method, or the industrial process from which lignin is derived, we can classify the type of lignin as follows:

1) Organosolv lignin: This lignin is obtained directly from biomass by extraction with organic solvents, i.e., soda lignin. The extraction process is a procedure for pulping non-wood materials, such as straw, sugarcane bagasse, or flax. Biomass is digested with aqueous solutions of sodium hydroxide at temperatures of 160° C or lower.
2) Steam-exploded lignin: This lignin is obtained from wood that is treated with steam at high temperature and high pressure, following a sudden decompression in the presence of acids, bases, and other chemicals to improve depolymerization.
3) Kraft lignin: It is the lignin obtained as a byproduct in the pulp and paper industry using sulfides in an alkaline environment with temperatures up to 170°C.
4) Sulfite lignin: It is the lignin that is also obtained in the pulp and paper industry in a process of biomass with SO_3_^2−^ salts or HSO_3_^−^, at a range of temperatures from 50 to 160°C.

The applications of the different types of lignin in a direct way have been extensively described in the literature (Calvo-Flores, 2020). For example, lignin is used in the preparation of resins, composites, polymers, carbon fibers, binders, and adhesives (Yue et al., 2017) (Solt et al., 2018). Furthermore, lignin can be also transformed into syngas, and thus, it can follow the syngas platform, previously described, to be converted in fuels or other valuable molecules.

Within the lignin platform, the current state-of-art in the catalytic processes aimed to the so-called lignin-first biorefinery, which is represented by the reductive catalytic fractionation (RCF), as described below.

d.1) Lignin-first biorefinery: Despite its potential and the existence of already developed applications, the greatest challenge in lignin-based technologies is the production of platform chemicals, especially those with aromatic functional groups such as benzene, toluene, and xylenes, i.e., BTX (Liu and Ragauskas, 2021). Reductive catalytic fractionation (RCF) is a promising lignin-first biorefinery strategy that yields a deeply depolymerized lignin and nearly theoretical amounts of lignin monomers with reductive catalysts (Qiu et al., 2020). The most essential ingredients to operate an RCF biorefinery are (i) lignocellulosic biomass, (ii) an alcohol (or cyclic ether) solvent, and (iii) a heterogeneous redox-active catalyst (Renders et al., 2019).

One of the main challenges for lignin valorization is the recondensation processes, i.e., new carbon–carbon bond formation that occurs during depolymerization in many of the most traditional fractionation processes, i.e., organosolv, kraft, and sulfite (Guadix-Montero and Sankar, 2018). Addressing this issue is crucial for the development of lignin valorization strategies. Moreover, most of the research focus has been traditionally placed on the beta-O-4 bond in the lignin structure (Sanchez-Gonzalez et al., 2017), but other relevant bonds need to be targeted as well.

**FIGURE 4:**
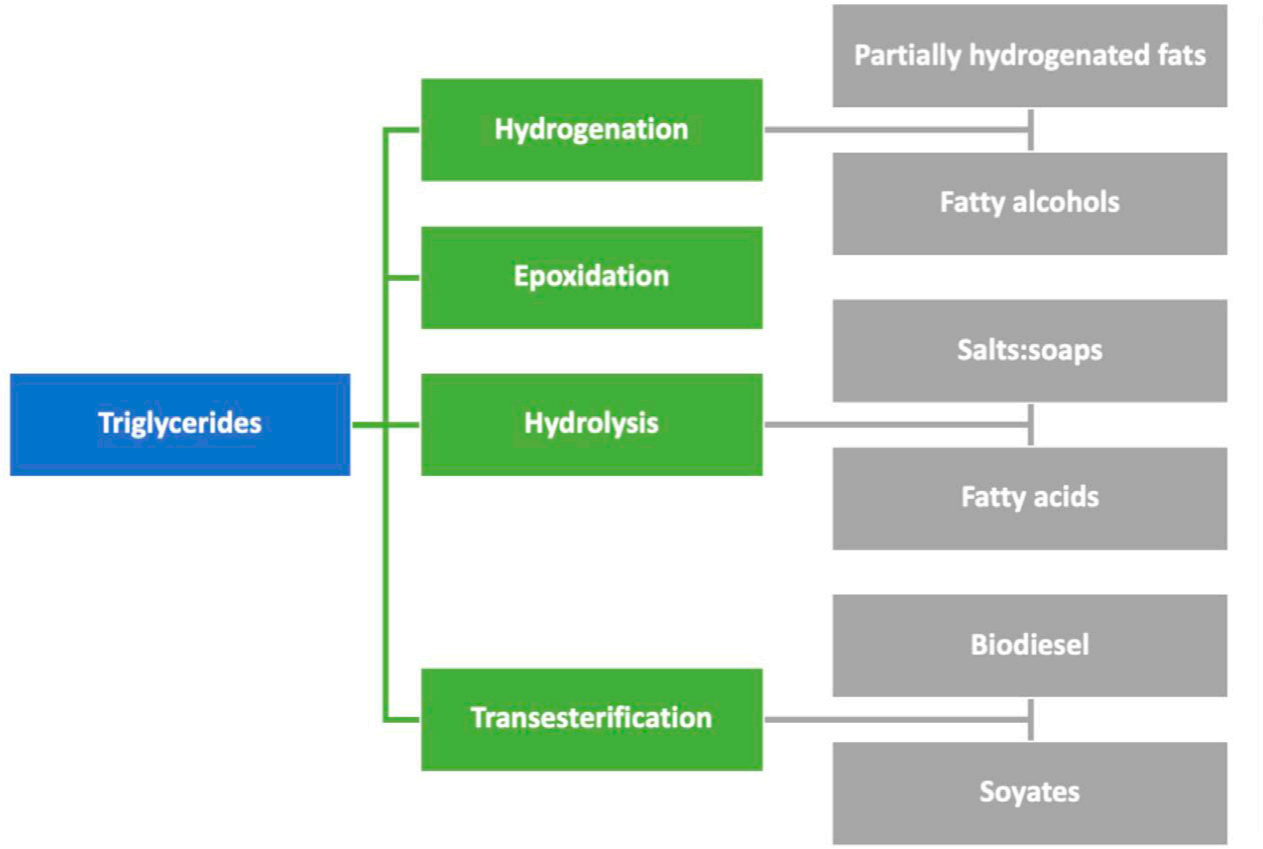
Schematic classification of the main triglycerides’ transformations (in green) and the main derivatives obtained (in gray).

e) Plant-based oil platform: Vegetable oils and fats are a wide group of renewable feedstocks with multiple applications across the chemical industry (Biermann et al., 2021). Oleochemistry is devoted to applications of oils and their derivatives for the preparation of valuable products, known as oleochemicals. The main sources of oils with no-feed applications are oils obtained from palm, soy, coconut, algae, and waste oils from food-related activities. Oleochemicals are widely used to produce biofuels, lubricants, coatings, adhesives, elastomers, sealants, household, and industrial cleaning, as well as in personal care products, pharmaceuticals, and nutraceuticals. The products obtained are cost- and performance-competitive with similar products from the petrochemical industry, while being non-toxic and environment-friendly (Tesser et al., 2020). Plant- and algae-based biorefineries are already producing this set of products (Caullet and Le Nôtre, 2015), with special focus on valorizing triglycerides, the main components of oils and fats (Figure 4). The major groups of products and chemicals from these oils are as follows:

1) Triglycerides: Partially hydrogenated triglycerides are extensively used in the food industry for the preparation of margarine and many other processed foods (Akoh, 2017). However, the consumption of these products is under suspicion because they have been associated with some heart diseases and other circulatory pathologies (Lawrence, 2013).
2) Fatty alcohols: These alcohols are obtained by hydrogenation of alcohols, and they have multiple applications. For example, they are extensively used as plasticizers in cosmetic and personal care products for the stabilization of emulsions and suspensions (Kelm and Wickett, 2017) (Majdzadeh-Ardakani et al., 2010). The sulfates derived from them are used to produce detergents and surfactants (Shampoo Formulation, 2007).
3) Epoxides: Vegetable oil–based epoxides are mostly produced on an industrial scale from soy oil. They have many industrial applications, for example, PVC stabilizers, plasticizers, components of bio-based resins, coatings, and lubricant formulations. Epoxidation is performed with peroxyacids (Prileschajew reaction) or through chemo-enzymatic reactions.
4) Fatty acid salts: Triglycerides are hydrolyzed to produce fatty acids and their salts. Salts of fatty acids are popular in home-made soap fabrication obtained when the process takes place under alkaline conditions (Kumar et al., 2016; Blonski, 2017). Free fatty acids may be synthetized by acidic hydrolysis, enzymatic conditions, or by selective oxidative cleavage of unsaturated triglycerides as in the case of pelargonic acid and nonanedioic acid (azelaic acid), valuable products for bio-based polymers and agrochemicals, obtained from sunflower oil with copper-based catalysts (Vassoi et al., 2021)
5) Triglyceride-derived biodiesel: The transesterification of triglycerides is specially focused on the production of biodiesel. Chemically, biodiesel is a mixture of methyl and/or ethyl esters of fatty acids obtained by transesterification of triglycerides and has become one of the more popular biofuels (Shahid et al., 2021). Biodiesel is synthetized by basic or acid catalysis (Shakorfow and Mohamed, 2020), or enzymatically (Ferreira and Tonetto, 2017); however, the simplest method is the classic basic catalysis. As a byproduct, glycerol is obtained in this reaction. In the context of an integrated installation, glycerol is a versatile building block (Mota et al., 2017) susceptible to be transformed into other valuable molecules (Takkellapati et al., 2018).

Within the plant-based platform, we have two main platform technologies:

a) Algae-based platform

Although most oils are obtained from seeds and fruits, in the last decades; there has been a growing interest in algae as a suitable biomass source for the extraction of valuable products. Algae can be produced on a large scale in all seasons with sunlight, CO_2_, nutrients, and water, constituting a remarkable area in the bioeconomy (Araújo et al., 2021; Ullmann and Grimm, 2021). Algae biomass can be relatively easily transformed in a biorefinery because of its lignocellulose-free nature. It is usually converted in a similar way than other plant-based biomass with thermochemical or biotechnological transformations. There are several possibilities for exploitation (Chew et al., 2017), for example, triglycerides for biodiesel production, fertilizers, food industry additives, nutritional supplements, or valuable molecules such as chlorophyll-a, phycocyanin, *ß*-carotene, or γ-linolenic acid, to mention a few (Laurens et al., 2017).

b) Press juice platform.

**FIGURE 5:**
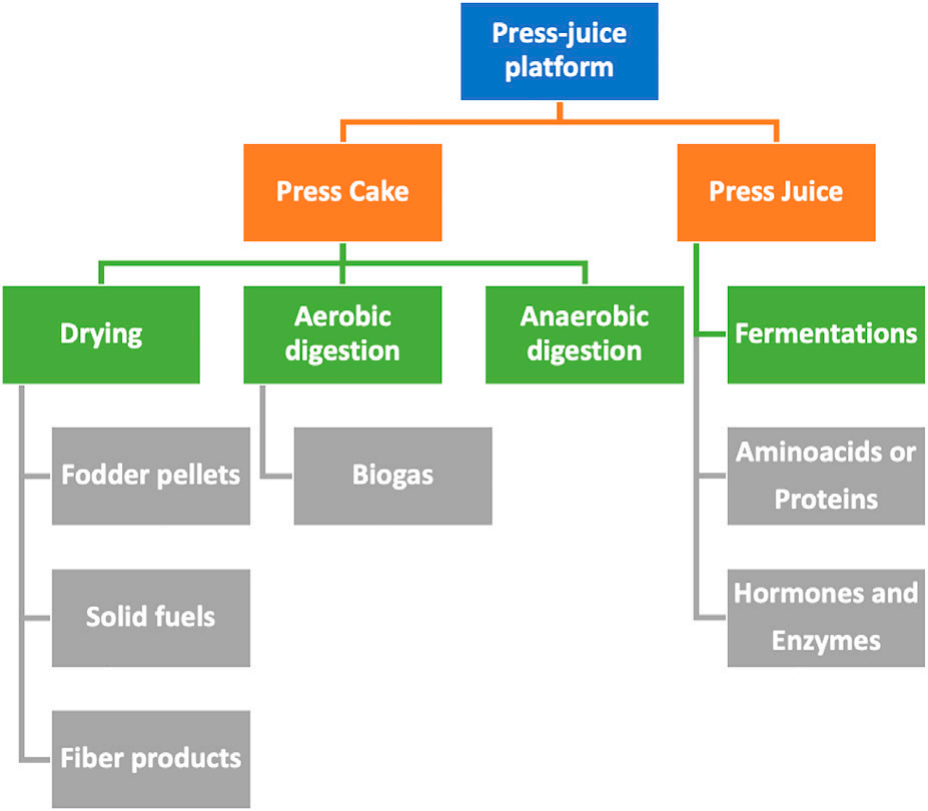
Schematic representation of the possible routes in the press juice (in blue). It includes the main intermediates (in orange) to produce valuable products (in gray), through different processes (in green).

This platform is based on the products obtained in the liquid phase after pressing wet biomass. The feedstock is green crops of the kind of alfalfa, clover, sugar beet leaf, potato leaf, or immature cereals. Biomass is pressed to obtain two streams (Thomsen, Andersen, and Kiel) (Figure 5):

1) A liquid extract containing a set of valuable substances, i.e., press juice. Amino acids, proteins, carboxylic acids, hormones, and enzymes are the common substances obtained in this liquid. The components of the liquid extract are separated using the different technologies, namely, decantation, drying, centrifugation, membrane filtration, purification, nanofiltration, electrodialysis, and chromatograph. The remaining supernatant or the components isolated by those techniques can be transformed later with microorganisms or enzymes (Thomsen, Andersen, and Kiel) (Lübeck and Lübeck, 2019).
2) The solid residue, named press cake, is a fiber-rich material that is also useful. It is formed by a mixture of lignin, cellulose, starch, dyes, pigments, and other organic molecules. It can be used as fuel or feed pellets. It can be also transformed through thermochemical conversion using gasification, pyrolysis, or hydrothermal liquefaction, or through anaerobic fermentation to produce biogas or fiber products, such as boards, insulation materials, horticultural substrates, and bio-composites.

## 3 Biorefinery products

### 3.1 Biofuels

Biofuels are any solid, liquid, and gaseous fuel produced from biomass (Van Ree et al., 2011), (Hingsamer and Jungmeier, 2019). The term also includes any pyrolysis oil that is used as fuel in boilers, engines, and turbines for heat and power generation (Pattiya, 2018). In addition to fuel applications (Review on bioethanol as alternative fuel for spark ignition engines, 2016), bioethanol is used as oxygenated additive in gasoline engines (Jafari et al., 2010) and also added as a gasoline octane booster instead of methyl-tert-butyl ether, which has been banned in many countries because of its environmental impact on natural and drinking waters (Kümmerer, 2011), (Calvo-Flores et al., 2018). In 2021, the total European Union (EU)’s bioethanol production for fuel reached roughly 5.6 billion liters (Fuel ethanol production in the EU, 2012–2021) and in the United States about 56 billion liters in the same period (Fuel ethanol production in the U.S. 1980-2021). Further expansion of first-generation bioethanol is expected to be limited. Expansion of second-generation cellulosic bioethanol production remains constrained due to high costs and a lack of certainty in the EU policy-making process, which is one of the challenges for second-generation biorefineries (fuel ethanol production in the U.S. 1980–2021). More specifically, the main types of biofuels currently being used or under development are

1) Biodiesel: A biofuel synthetized from oils by transesterification reactions. It is one of the most popular biofuels, and it can be prepared from both dedicated and waste oils. The EU is the largest biodiesel producer worldwide, with a production in 2020 of around 3.4 million metric tons (Global HVO biodiesel production by country 2020).
2) Bioethanol: Ethanol obtained from biomass is commonly referred to as bioethanol. It can be obtained from several biomass sources through fermentation procedures, and it has become one of the most accessible biofuels. It can be used directly or blended with gasoline without substantial modification of car engines. The skyrocketing gasoline prices have increased the competitiveness of bioethanol. The volume of sugar beets as feedstock for ethanol is particularly increasing, as cereals such as wheat, are being prioritized for use as food or feed (Fuel ethanol production in the EU 2012–2021). On the other hand, bioethanol is a remarkable C2 building block. More discussion on this aspect will be provided later.
3) Biogas: A biofuel mostly composed of methane obtained from biomass by anaerobic fermentation. Biomass that breakdown under such conditions generates a mixture primarily consisting of carbon dioxide and methane in an average proportion that ranges between 50% and 85% (Sindhu et al., 2019). Biogas can be used directly in boilers for heat generation, incorporated into the natural gas distribution network, and transformed biotechnologically.
4) Hydrogen: The cleanest fuel because when consumed in a fuel cell, it produces only water as an oxidized byproduct. In addition, it is a very efficient energy carrier for storing, moving, and delivering energy produced from other sources. Current hydrogen production occurs from fossil fuels or is produced by splitting water. When renewable energies are used in the splitting water, the hydrogen produced is usually referred to as green hydrogen. A novel option for hydrogen preparation based on lignocellulosic gasification is currently in a pilot-scale demonstration stage (Lepage et al., 2021). Furthermore, catalyzed aqueous phase reforming (APR) of various oxygenated hydrocarbons derived from biomass, such as ethanol, ethylene glycol, polyols (glycerol, sorbitol, and glucose), cellulose, and woody biomass, is a promising process to produce hydrogen under wet conditions. In APR, hydrocarbons or oxygenates are dissolved in water and react with water molecules at low temperatures and high pressures to form hydrogen and hydrocarbons (Li He and Yang, 2013).
5) Biomass pellets: These are probably the simplest fuel obtained from biomass. Generally, they are made from several sources such as wood and agricultural wastes, commercial grasses, forestry residues, and nowadays from almost all residual biomasses. They are easy to store, and transport, and can be produced in most biorefineries (Frodeson et al., 2019). Pelletization is one of the most popular biomass exploitations for domestic heating, despite wood.

### 3.2 Chemical building blocks and bio-based solvents

The chemical industry produces thousands of substances that are present in our everyday life directly or indirectly. Most of these molecules are synthesized in a petrochemical-based technology, although they can potentially be produced in biorefineries. Functionalized molecules, usually with more than one functional group, as well as the basic building blocks from biomass can be used in organic synthesis to produce more complex molecules or to prepare bio-based solvents.

One of the first attempts to systematically analyze the viability of products that are obtained from biorefineries as an alternative to those obtained from the petrochemical industry was carried out in 2004 by the U.S. Department of Energy (DOE). The DOE identified a group of 30 bio-based chemical building blocks (Werpy and Petersen, 2004) from an initial screening of almost 50 substances (Table 2). The report started to be implemented a few years later, including lignin as an option for the obtention of new compounds of interest (Holladay et al., 2007).

**TABLE 2:** Bio-based chemical building blocks as identified by the U.S. Department of Energy in 2004.

Label based on the number of carbons	Potential top 30 candidates
C1	Carbon monoxide and hydrogen (syngas)
C2	Discarded
C3	Glycerol, 3-hydroxypropionic acid, lactic acid, malonic acid, propionic acid, serine
C4	Acetoin, aspartic acid, fumaric acid, 3-hydroxybutyrolactone, malic acid, succinic acid, threonine
C5	Arabinitol, furfural, glutamic acid, itaconic acid, levulinic acid, proline, xylitol, xylonic acid
C6	Aconitic acid, citric acid, 2,5-furandicarboxylic acid, glucaric acid, lysine, levoglucosan, sorbitol

In a following report in 2007 (Holladay et al., 2007), this initial group was further reduced to 12 substances that were considered preferential because of their versatility as synthetic building blocks, and their competitive cost compared to those of petrochemical industry alternatives (Table 3).

**TABLE 3:** Selected bio-based building blocks.

Selected building block	
1,4-Diacids (succinic, fumaric, and malic)	Itaconic acid
2,5-Furan dicarboxylic acid	Levulinic acid
3-Hydroxypropanoic acid	3-Hydroxybutyrolactone
Aspartic acid	Glycerol
Glucaric acid	Sorbitol
Glutamic acid	Xylitol/arabinitol

Based on the status of the 12 chemicals listed in 2004 (Choi et al., 2015), and considering a further revision that took place in 2010 (Bozell and Petersen, 2010), it is a fact that most of these building blocks have been commercialized or are on their way to commercialization.

#### 3.2.1 C1 building blocks: carbon dioxide

**FIGURE 6:**
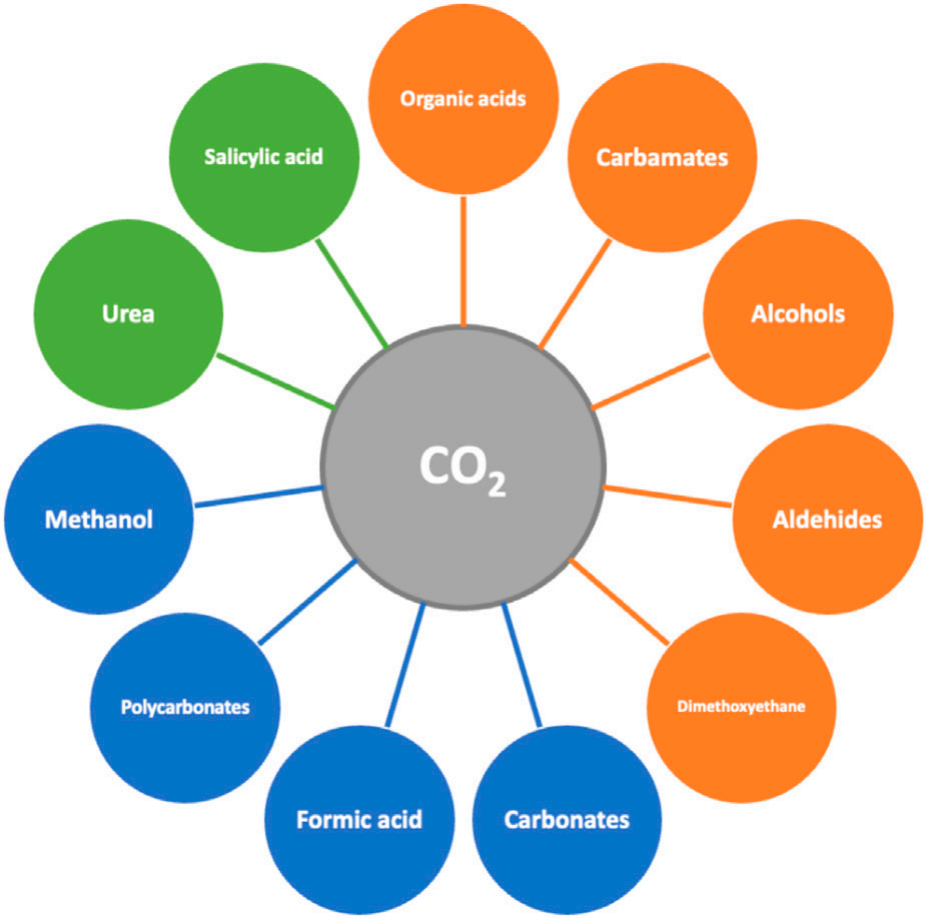
Valuable products from carbon dioxide, color-coded according to the current stage of development in their production, i.e., laboratory scale (orange), demonstration plant (blue), and commercial stage (green).

These first approaches to identifying key bio-based building blocks did not consider carbon dioxide as one alternative, despite being one of the simplest carbon sources. It is well-known that carbon dioxide is a greenhouse gas formed in all combustion processes, as well as in aerobic fermentation. It is, therefore, a biogenic product whose valorization can be integrated into biorefinery systems. The valorization of products that are obtained from carbon dioxide is at different stages of development. The production of organic acids, carbamates, alcohols, aldehydes, and dimethoxyethane (DME) is in the laboratory scale. There are demonstration plants to produce methanol, formic acid, carbonates, and polycarbonate, while only some products such as urea and salicylic acid are being commercialized. Figure 6 summarizes the possible valuable products that can be produced out of carbon dioxide, and the current stage of development in their production (Dahmen et al., 2017).

#### 3.2.2 C2 building blocks: ethanol

As it happened with carbon dioxide, C2 compounds such as ethanol, and its derivatives such as acetic acid and acetic anhydride, were initially discarded as well, in the first classifications. Their easy availability from the petrochemical industry made them of low interest as potential compounds to be extracted from biomass sources. The scenario has changed, and bioethanol (Edeh, 2021) has become one of the most relevant bio-based compounds, not only because of its application as fuel but also due to its potential as raw material for subsequent transformations into other platform chemicals and solvents (Aeschelmann et al., 2017; Baltus, 2017), (Eswari et al., 2020), (Galanakis, 2020).

**SCHEME 1:**
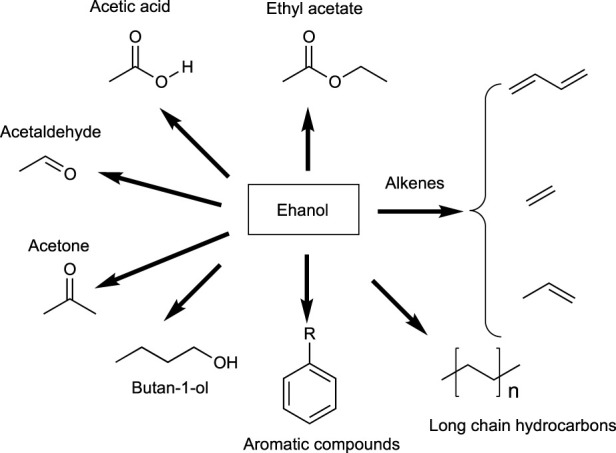
Valuable products that can be chemically derived from ethanol.

Scheme 1 depictures a set of substances traditionally obtained from ethanol in the petrochemical industry (Calvo-Flores et al., 2018), and therefore the potential bio-based chemicals that can be produced through the same known routes but starting from bioethanol as raw material. Table 4 summarizes the uses and applications of these bioethanol derivatives.

**TABLE 4:** Uses and applications of bio-derivatives from ethanol.

Product	Use
Ethyl acetate	Degradable solvent of general uses
Butadiene	Production of synthetic rubber shoe soles, tyres, etc.
Ethene (ethylene)	Polymers, preparation of numerous chemicals, phytohormone
Propene (propylene)	Polymers, preparation of numerous chemicals
Long chain hydrocarbons	Waxes
Aromatic compounds	Raw materials for numerous chemicals
Butan-1-ol	Environmentally friendly solvent, chemical intermediate for numerous chemicals
Acetone	Multipurpose environmentally friendly solvent
Acetaldehyde	Manufacture of acetic acid, perfumes, dyes and drugs, as a flavoring agent
Acetic acid	Reaction media, reagent for the production of chemical compounds

#### 3.2.3 C3 building blocks: glycerol and lactic acid

**SCHEME 2:**
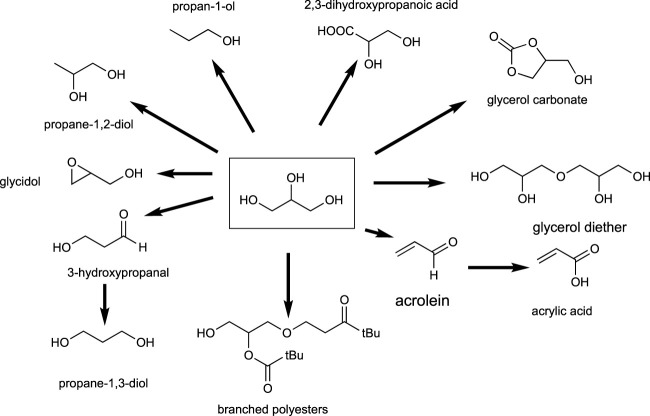
Possible glycerol transformations into valuable products.

Glycerol is a non-toxic substance of natural occurrence that is obtained in great quantities as a byproduct in the biodiesel production from vegetable oils. It is not only a byproduct but also a building block suitable of a multitude of transformations to produce many valuable chemical compounds (Posada et al., 2012) (D’Angelo et al., 2018). Scheme 2 depicts the molecules and families of molecules that can be produced from glycerol. The variety of possible functional groups, e.g., alcohols, acids, and ethers, makes this building block of special interest for the synthesis of monomers in the polymer industry. Table 5 summarizes the key uses and applications of bio-based chemicals from glycerol.

**TABLE 5:** Uses and applications of bio-derivatives from glycerol.

Product	Use and application
2,3-Dihydroxypropanoic acid	Multifunctional monomer, surfactant
Glycerol carbonate	Polar solvent, synthesis of polymers, surfactants
Glycerol diether	Preparation of fatty acid esters, as emulsifiers and defoamers for printing
Acrolein	Biocide, precursor of many chemicals, synthesis of polymers
Branched polyesters	Pharmaceutical and biomedical applications, dendritic materials
Propane-1-3-diol	Solvent, humectant, antifreeze, difunctional monomer
3-Hydroxypropanal	Food preservation, as a precursor for many chemicals such as acrolein, acrylic acid, and 1,3-propanediol
Glycidol	Stabilizer in manufacturing of vinyl polymers, intermediate in synthesis of glycerol derivatives, additive for oil and synthetic hydraulic fluids, epoxy resin diluent
Propane-1,2-diol	Coffee-based drinks, liquid sweeteners, ice creams, antifreeze, solvent in e-cigarettes
Propan-1-ol	Multipurpose solvent

**SCHEME 3:**
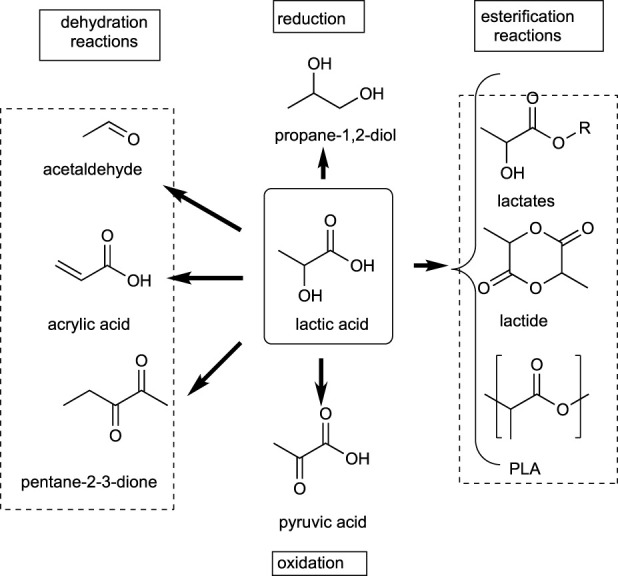
Valuable molecules that can be derived from lactic acid.

Another C2 building block of interest is lactic acid. It is a versatile compound for the synthesis of many fine chemicals (Datta and Henry, 2006), and it is used as a monomer for the synthesis of biocompatible polymers (Sin and Tueen, 2019) (Abedi and Hashemi, 2020)). Moreover, lactic acid and their ethyl, propyl, and butyl esters are considered as green solvents (Yang et al., 2012), which provides an additional avenue for the valorization of this bio-based building block. Especially popular is its ethyl ester (Paul et al., 2016), which is used to replace other more environmentally harmful solvents such as acetone, xylene, methyl ethyl ketone (MEK), and toluene (Pereira et al., 2011). Additionally, using current biotechnological processes it is possible to obtain racemic or enantiomerically pure R or S isomers of lactic acid (Abedi and Hashemi, 2020), which provides additional versatility when looking for applications. Furthermore, it is the starting material of many other molecules available from the petrochemical industry, such as acrylic acid or acetaldehyde, and monomers for the synthesis of biodegradable polymers such as polylactates. Scheme 3, describes the main derivatives obtained from lactic acid, grouped according to the reaction type: dehydration, oxidation, reduction, and esterification. Table 6 describes the key uses and applications of these bio-based chemicals from lactic acid.

**TABLE 6:** Uses and applications of bio-derivatives from lactic acid.

Product	Use and application
Propane-1,2-diol	Coffee-based drinks, liquid sweeteners, ice creams, antifreeze, solvent in e-cigarettes
Lactates	Green solvents
Lactide	Monomer for poly lactate synthesis
Polylactates (PLA)	Biodegradable implants and devices, packaging films, containers, food service wares, and bottles with short shelf life
Pyruvic acid	Reagent for organic synthesis, sold as weight-loss supplement
Pentane-2-3-dione	Solvent, flavor, synthesis of dyes, pesticides, drugs
Acrylic acid	Synthesis of polymers, production of hygienic medical products, detergents, and wastewater treatment chemicals
Acrolein	Biocide, precursor of many chemicals, synthesis of polymers

#### 3.2.4 C4 building blocks: succinic acid

**SCHEME 4:**
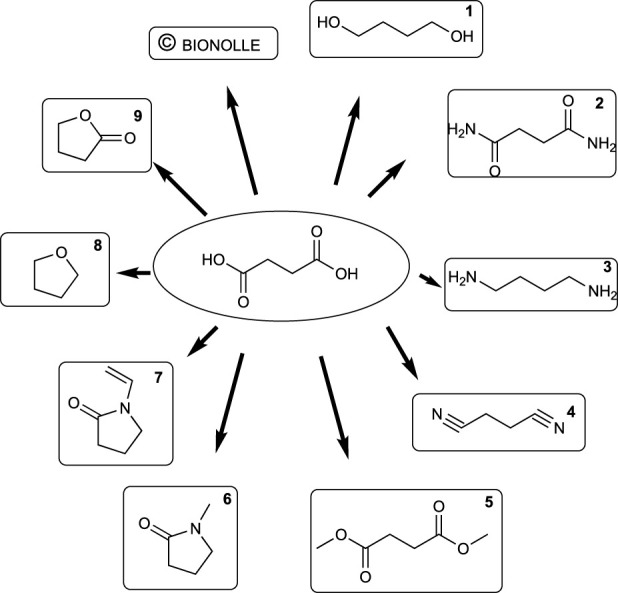
Valuable products that can be derived from succinic acid.

Succinic acid is a C4 building block that can be also obtained from biomass sources (Delhomme et al., 2009). It has been used as a starting molecule for the synthesis of many commodity chemicals during the last decades (Werpy, Frye, and Holladay), and therefore bio-based succinic acid and its derivatives are now used for producing similar relevant products, such as excipients in pharmaceutical preparations, surfactants, detergents, flavoring agents, and fragrances. It is also the precursor of some biodegradable polymers, polyurethanes, polyethers, and polyesters in clothing fibers. In the food industry and in agriculture, it is used as an acidity regulator, fungicide, and herbicide (Saxena et al., 2017). Scheme 4 describes some key chemicals that can be prepared from succinic acid, and their main applications are summarized in Table 7.

**TABLE 7:** Uses and applications of bio-derivatives from succinic acid.

Number	Name	Use and application
1	Butan-1,4-diol	Difunctional monomer
2	Succinamide (butanediamide)	Difunctional monomer
3	Butan-1,4-diamine	Polyamide synthesis
4	Succinonitrile	Starting materials for polymers
5	Dimethyl succinate	Versatile building block, flavoring agent, additive for paints and coatings
6	N-methylpyrrolidone	Solvent for pharmaceutical industry, textile surface treatment, photo resistant stripping agent
7	N-vinylpyrrolidone	Precursor to polyvinylpyrrolidone (povidone)
8	Tetrahydrofuran	Multipurpose solvent
9	gamma-Butyrolactone (GBL)	Biofuel, fuel additive, solvent
	Bionolle	Polybutylene succinate, green alternative to polyethylene

#### 3.2.5 C5 building blocks: levulinic acid

**SCHEME 5:**
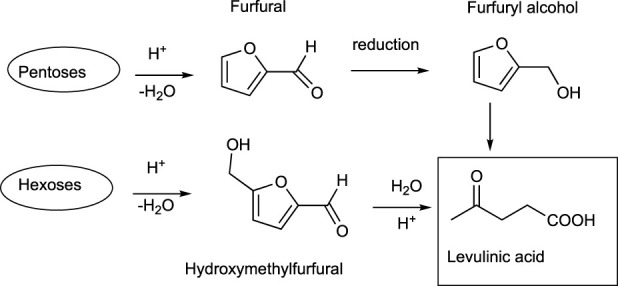
Synthetic routes for levulinic acid from pentoses (C5) and hexoses (C6).

Bio-based levulinic acid is produced from pentoses (C5 sugars) and hexoses (C6 sugars), although the synthesis from glucose, through 5-hydroxymethylfurfural (HMF) as an intermediate, is the main route (Scheme 5) (Kumar et al., 2020).

**SCHEME 6:**
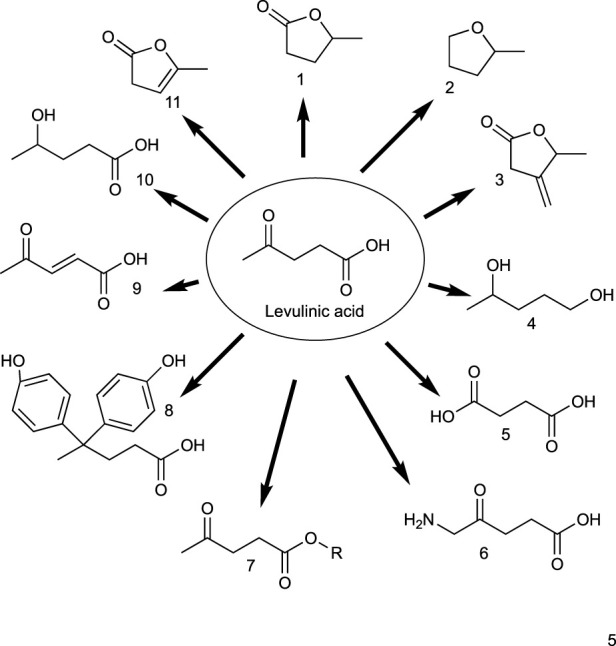
Valuable products that can be produced from levulinic acid.

Levulinic acid is the starting material for several valuable products in the the pharmaceutical industry, agrochemicals, food additives, fragrances, personal care commodities, solvents, and polymers. Scheme 6 depicts some levulinic acid derivatives, while Table 8 summarizes the uses and applications of bio-based compounds from succinic acid. Despite its potential and proven applications, the lack of high yields in the preparation of levulinic acid from biomass feedstock is still hampering its scale-up and commercialization. Further developments across the different platform technologies are still needed to produce levulinic acid in a cost-competitive way (Dutta and Bhat, 2021).

**TABLE 8:** Uses and applications of bio-derivatives from levulinic acid.

Number	Name	Use
1	γ-Valerolactone	Biofuel, fuel additive, solvent
2	2-Methyltetrahydrofuran	Biofuel, fuel additive, solvent
3	2-Methylene-γ-valerolactone	Biofuel, fuel additive
4	1,4-Butanediol	Solvent, monomer for polyesters, organic synthesis
5	Succinic acid	Monomer, synthesis of pesticides, solvent
6	Amino lactic acid	Cancer treatment, agrochemicals
7	Levulinic acid esters	Solvents, fuel additives, plasticizers, food flavoring
8	4,4-Bis(4-hydroxyphenyl) pentanoic acid	Monomer, adhesives, lubricants
9	Acetyl acrylic acid	Monomer
10	Hydroxy valerianic acid	Fuel additive, paints, resins
11	5-Methylfuran-2(3H)-one	Fuel additive, monomer, solvent

#### 3.2.6 C6 building blocks: 5-hydroxymethylfurfural and 2,5-furandicarboxylic acid

**SCHEME 7:**
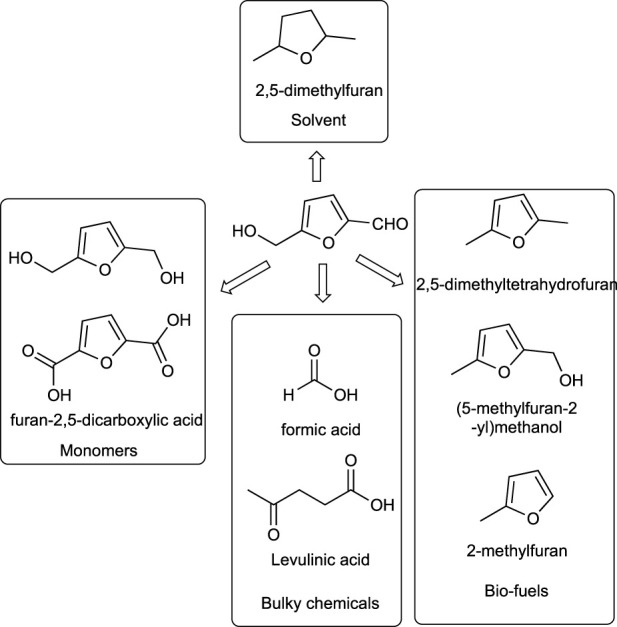
Building blocks from 5-hydroxymethylfurfural.

**SCHEME 8:**
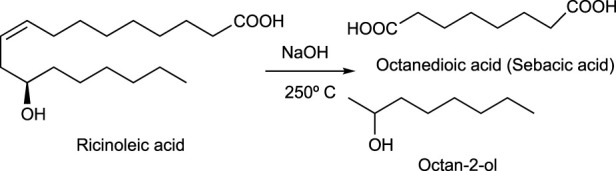
Main reaction in octan-2-ol (2-octanol) synthesis.

HMF is a key biomass platform intermediate that is commonly obtained from glucose or fructose by dehydration reactions. Further chemical reactions of HMF give a set of valuable organic molecules to be used as chemical building blocks (Scheme 7) (Boisen et al., 2009) (Wang et al., 2018). Scheme 8 shows the main products that can be obtained from HMF and their uses. Among them, 2,5-furandicarboxylic acid (FDCA) is currently recognized as one of the most important bio-based synthons due to its potential application as a monomer to synthesize green polymers. Thus, FDCA has become an ideal green alternative to terephthalate (Drault et al., 2020), which is used in the synthesis of one of the most popular thermoplastic polyesters (Intratec, 2017). The classical synthesis of FDCA is by the oxidation of HMF with oxidants such as KMnO_4_ (van der Klis et al., 2017), which is obtained from monosaccharides by dehydration reactions.

### 3.3 Bio-based polymers

Polymers synthetized from biomass, bio-based polymers, are a more sustainable alternative to conventional polymers traditionally prepared from petroleum, and they constitute a permanent growing market (Global biopolymers market to witness a CAGR of 15.2% during 2018–2024: Energies Market Research Pvt Ltd, 2018). Biorefineries are adapted to directly produce such polymers or to provide the starting monomers for their preparation using traditional routes. Most of the biopolymers are biodegradable, and in many cases biocompatible, which makes them to be considered as green polymers (Scott, 2000). They can be classified into two major groups:

a) Modified natural polymers from starch, cellulose, or lignin:
  1) Starch-based polymers: They are a family of biodegradable polymers (Jiang et al., 2020) with many applications, especially with the growing banning of disposable plastics. The fabrication of composites and blends based on starch-based polymers is one of the growing strategies for improving sustainability in packaging materials.
  2) Modified cellulose: Native cellulose can be modified through chemical reactions on its hydroxyl groups. The main reactions include oxidation, esterification, etherification, amidation, or through non-covalent modifications. In addition, cellulose-based composites can be prepared by blending cellulose derivatives with native cellulose (Shaghaleh et al., 2018) to provide a series of biodegradable materials. Among them, cellulose diacetate and cellulose triacetate are the most applied, with numerous industrial applications in textiles such as fibers and threads for quality fabrics; in plastic films such as optical film for LCD technology or antifog goggles; and in many other consumer products such as cellulose based filters, window cartons, or labels (Rustemeyer, 2004; Heinze and Liebert, 2012).
  3) Lignin-based polymers: These polymers can be either prepared from unmodified native or industrial lignins, or from chemically modified lignin. Both lignin-based polymers are the starting material for composites (Glasser, 2016), bio-based nanomaterials (Grossman and Vermerris, 2019), and carbon fibers (Wei and Oksman, 2022).
b) Polymers from biomonomers: Monomers from renewable sources that are used in polymer chemistry are commonly referred to as biomonomers (Visakh, 2019). The polymers can be synthesized from a single difunctional biomonomer molecule such as dialcohols to give polyethers, or from hydroxy acids to give polyesters. They can be also formed by the combination of two different difunctional biomonomers. For example, carboxylic diacids and dialcohols to give polyesters or carboxylic diacids and diamines to give polyamides (Rebouillat and Pla, 2016). Examples of polymers from combinations of biomonomers are as follows:
  1) Polyesters derived from
    a) Succinic acid, lactic acid, and hydroxy acids
    b) Diols such as 1,3-propanediol and 1,4-butanediol
  2) Furan-based polyesters from HMF:
    a) Polyamides combining amino acids, lactams, dicarboxylic acid, and diamines
    b) Styrenic vinyls polymers from p-hydroxystyrene (pHS) and styrene from glucose (McKenna and Nielsen, 2011)

### 3.4 Bio-based solvents

Organic solvents are used in all areas of industry, laboratories, and consumer products. They have a well-known significant toxicity, imply a relevant environmental impact, and they are mostly prepared from non-renewable sources. Thus, biorefineries provide a more sustainable alternative also to produce green solvents that are eco-friendly and less toxic than conventional ones (Clarke et al., 2018).

#### 3.4.1 Alcohols

**SCHEME 9:**
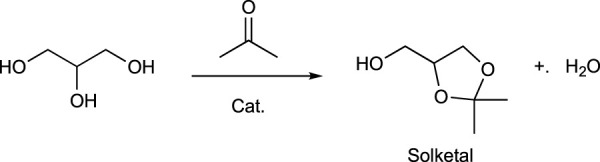
Synthesis of solketal.

Bioethanol obtained from biomass is a well-established fuel, raw material, and solvent, as described. It can be prepared from several feedstocks including cellulose, hemicellulose, or starch. In fact, sugarcane, sugar beets, corn, molasses, potatoes, wheat, barley, oat, rice, and even lignocellulosic materials from agricultural wastes or woody biomass can be transformed into bioethanol (CHAPTER 6: An Appendix of Solvent Data Sheets, 2017). Some examples of bio-based alcohols are as follows:

a) Butan-1-ol (1-butanol) is obtained from biomass through fermentation using an acetone-butanol-ethanol (ABE) process with clostridia bacteria and various commercial raw materials such as molasses, whey permeates, corn, and lignocellulosic biomass. In the last few years, the biotechnological process to produce butan-1-ol (1-butanol) has been improved and it is economically equivalent to better-established petrochemical processes (Ndaba et al., 2015).
b) Octan-2-ol (2-octanol) is a C8 fatty alcohol manufactured by a cracking process of ricinoleic acid, a major component of castor oil (CHAPTER 6: An Appendix of Solvent Data Sheets, 2017). It can be produced from bio-based ricinoleic acid (Scheme 8).
c) Bio-based 1.3-propanediol (PDO) is manufactured by DuPont through a proprietary fermentation process that uses plant-derived glucose instead of petroleum feedstocks (Our Process, 2019).
d) Butane-1,3-diol, also named as 1,3-butylene glycol, can be prepared from molasses (Jiang et al., 2014).
e) Glycerol has been also explored as a green solvent (Díaz-Álvarez et al., 2011) (Gu and Jérôme, 2010) because of its biocompatibility and its almost zero toxicity. It can be used in many pharmaceuticals and cosmetic formulations, although its use as a reaction medium is limited because of its high boiling point (290°C), which makes it difficult to remove it from crude reactions. The isopropylidene acetal derivative of glycerol, known as solketal (Aldea et al., 2010) is used as fuel additive (Nascimento et al., 2019) (Scheme 9).

#### 3.4.2 Lactic acid and its esters

As discussed before, lactic acid is a valuable molecule, used not only as a monomer for the synthesis of biodegradable polymers but also as a solvent in many personal care products (Dusselier et al., 2013) and as an alternative solvent to conventional ones in many organic reactions (Yang et al., 2012). As commented before, ethyl and propyl lactic esters are produced from biomass sources, both the S or R isomers depending on the microorganisms used in the process. These lactic acid esters can be used as bio-based solvents, replacing more harmful organic solvents such as acetone, xylene, methyl ethyl ketone (MEK), and toluene (Pereira et al., 2011).

#### 3.4.3 Carbohydrate derivatives

**SCHEME 10:**
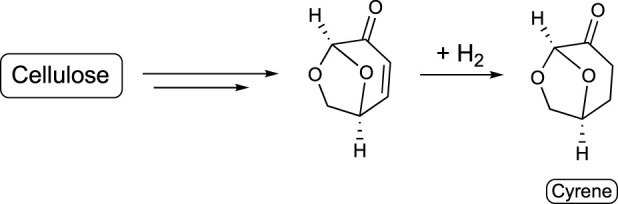
Synthesis of cyrene.

**SCHEME 11:**
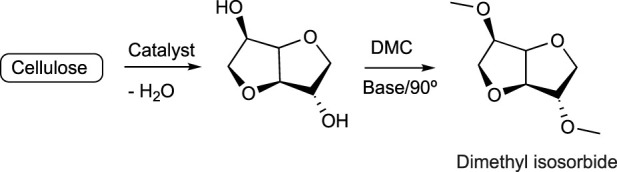
Synthesis of dimethyl isosorbide.

**SCHEME 12:**
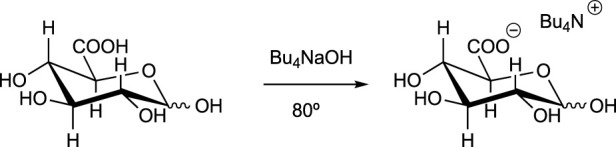
Example of a bio-based ionic liquid.

Bio-based solvents derived from carbohydrates have been also developed. Some examples are as follows:

a) Dihydrolevoglucosenone (Cyrene): This is a commercially available bio-based solvent that is produced by pyrolysis of lignocellulosic biomass, and specifically from cellulose (Kong and Dolzhenko, 2022) (Scheme 10). It can replace toxic dimethyl formamide or dimethyl sulfoxide (Camp et al., 2020) as a reaction medium for many chemical reactions, including polymerizations.
b) Dimethyl isosorbide: This is a dipolar aprotic solvent commercially available from cellulose (Tundo et al., 2010) (Scheme 11). It is commonly used as reaction medium, co-solvent, and also to improve the solubility of nonpolar compounds, or in the preparation of polymer membranes.
c) Carbohydrate-based ionic liquids: These are a subclass of ionic liquids (Qureshi et al., 2014) formed by at least one moiety of carbohydrates (Jopp, 2020). They constitute a promising group of solvents with applications in organic synthesis, extraction of natural products, and production of biocompatible materials. An example of the production of one of these compounds is described in Scheme 12.

#### 3.4.4 Other bio-based solvents

In addition to the main classes of solvents already discussed, there are other bio-based compounds that are being used in solvent applications. Some examples are as follows:

a) γ-Valerolactone (GVL): It is known to be an excellent bio-based solvent, which, today, can be prepared from levulinic acid (Table 8). It is significantly less hazardous to humans than various organic solvents such as NMP, DMAc, or DMF, showing similar and often lower ecotoxicity so it may be used in the food industry as a flavoring agent (Kerkel et al., 2021)
b) d-Limonene: This is a natural product, present in orange peels, which is commercially obtained from peel wastes (Siddiqui et al., 2022). It is a substance capable of replacing toxic solvents such as toluene, n-hexane, and chlorinated organic for extraction processes (Ciriminna et al., 2014), as well as in cleaning and degreasing activities (Paggiola et al., 2016). In addition, it is a good candidate to produce polymers and other valuable products (Ciriminna et al., 2014).
c) Methyl soyates: These are biodiesel molecules utilized as industrial solvents and cleaners or degreasers for removing dirt, dust, and grease (Anna and Wypych, 2014).

### 3.5 Proteins, vitamins, dietary supplements, and human and animal feed

Agro-industrial byproducts are mostly derived from processing biomass. They contain a variety of valuable products such as sugars, minerals, proteins, vitamins, and pigments, which makes them of high value in biorefining processes (Philippini et al., 2020).

In the case of proteins, traditional techniques of extraction are often not useful for protein-rich biomass, due to the extreme conditions required, which increases the risk of undesirable reactions (De Schouwer et al., 2019). Current examples of the integration of protein extraction methods in the biorefinery of agri-food residues are basically at a laboratory scale or using conceptual designs. Their large-scale feasibility still requires further research (Contreras et al., 2019).

### 3.6 Fertilizers

The production of bio-based fertilizers and additives for farming and gardening is a safe alternative to valorize biological wastes across different biorefinery platforms (Chojnacka et al., 2020). Recently, the fertilizer replacement value (FRV) is an indicator of the suitability of a new bio-based fertilizer to substitute current chemical ones. Although organic fertilizers from biorefineries are quite easy to prepare, recovering inorganic nutrients with current technologies is challenging. Anaerobic digestion is becoming a good alternative to mineralize nutrients in biorefinery wastes (Adani et al., 2020). In fact, some studies have shown digestates with an N:P:K ratio similar to mineral fertilizers (Santamaria-Fernandez et al., 2020). Recovering agricultural nutrients from biorefineries, for a substitution of conventional fertilizers is a challenge for the next few years (Carey et al., 2016).

## 4 Conclusion

Biomass is meant to be the most critical source of carbon materials, as our economy gradually abandons coal and crude oil for the extraction of raw materials and fuels. The new bio-based economy grows in complexity, alongside higher crude oil prices, environmental concerns, corporate commitments, policy, and legislation. Many fuels, chemical building blocks, polymers, and solvents are already being produced from biomass and biomass waste in the new generations of biorefineries, and this production is expected to grow as the biorefinery system reaches the industrial scale. However, the cost of bio-based production still exceeds the cost of its petrochemical alternative, and bio-based products need to be proven to outperform their petrochemical counterparts. Moreover, this growth of a bio-based economy also implies an increase in feedstock demand, technology development, and business opportunities.

Nowadays, biorefineries have achieved enough degree of development to start providing reliable alternatives to most of the products needed in our daily day life. However, our biorefinery system is not yet at the point of displacing existing fossil refinery technologies. In fact, rather than displacing or even replacing existing petrochemical platforms, the biorefinery systems should aim at integrating with current infrastructure. The development of integrated biorefinery processes that produce both bio-based products and energy carriers is the most efficient strategy to valorize biomass in the future bio-based economy. The existence of several biorefinery platforms under development and the co-existence of competitive technologies are promising. Although some of these alternatives might not be successful in the long run, the fact that several options coexist only increases the chances of having full scale-up integrated biorefineries in the future.

Among several challenges ahead, it is necessary to improve many of the processes of biomass transformations to make them economically feasible in a continuous scaled-up operation. Additionally, it is necessary to develop new processing technologies, catalysts, and chemical reactions that enable the synthesis of substances that are not currently achievable by other means. In the case of carbohydrate-containing biomass, the current technological developments can perform almost anything, but the same does not apply to the lignin platform. For lignin, neither chemically nor biotechnologically platforms provide satisfactory results, despite lignin being the most abundant source of aromatic moieties in nature. Enabling a scaled-up lignin platform to produce aromatic molecules is probably one of the most important challenges for the next decade in this field. This is part of a greater challenge, which is to arrive at carbon-neutral industrial processes. To achieve this objective, biorefineries must consume renewable energy to reduce their carbon footprint, which implies a dialogue between several stakeholders and a strong push for system integration with other sectors and relevant technologies.
